# Speeding up tandem mass spectrometry-based database searching by longest common prefix

**DOI:** 10.1186/1471-2105-11-577

**Published:** 2010-11-25

**Authors:** Chen Zhou, Hao Chi, Le-Heng Wang, You Li, Yan-Jie Wu, Yan Fu, Rui-Xiang Sun, Si-Min He

**Affiliations:** 1 Key Lab of Intelligent Information Processing, Chinese Academy of Sciences, Beijing 100190, China; 2 Institute of Computing Technology, Chinese Academy of Sciences, Beijing 100190, China; 3 Graduate University of Chinese Academy of Sciences, Beijing 100049, China

## Abstract

**Background:**

Tandem mass spectrometry-based database searching has become an important technology for peptide and protein identification. One of the key challenges in database searching is the remarkable increase in computational demand, brought about by the expansion of protein databases, semi- or non-specific enzymatic digestion, post-translational modifications and other factors. Some software tools choose peptide indexing to accelerate processing. However, peptide indexing requires a large amount of time and space for construction, especially for the non-specific digestion. Additionally, it is not flexible to use.

**Results:**

We developed an algorithm based on the longest common prefix (ABLCP) to efficiently organize a protein sequence database. The longest common prefix is a data structure that is always coupled to the suffix array. It eliminates redundant candidate peptides in databases and reduces the corresponding peptide-spectrum matching times, thereby decreasing the identification time. This algorithm is based on the property of the longest common prefix. Even enzymatic digestion poses a challenge to this property, but some adjustments can be made to this algorithm to ensure that no candidate peptides are omitted. Compared with peptide indexing, ABLCP requires much less time and space for construction and is subject to fewer restrictions.

**Conclusions:**

The ABLCP algorithm can help to improve data analysis efficiency. A software tool implementing this algorithm is available at http://pfind.ict.ac.cn/pfind2dot5/index.htm

## Background

Database searching has become the key technology for shotgun proteomics. Many algorithms and software tools exist for such searches, including SEQUEST [1], MASCOT [2], X!Tandem [3], OMSSA [4], Phenyx [5], PepSplice [6], Crux [7] and pFind [8-10]. However, the existing tools are not quick enough, for the following reasons:

First, the size of protein databases is increasing significantly, resulting in many peptides. In addition, semi- or non-specific digestion generates 10 to 100 times more peptides than full-specific digestion. For example, the size of the all-species NCBInr protein sequence database was 3.7 GB in December 2008 and increased to 5.7 GB in June 2010. The number of non-redundant peptides generated by full-specific digestion with up to two missed cleavage sites in the IPI-Human V3.65 database [11] is 3549956, and it increases 170-fold to 626871441 for non-specific digestion.

Second, identification of peptides with chemical and post translational modifications requires much more time. The number of peptides in the IPI-Human database generated by full-specific digestion with up to two missed cleavage sites increases 37.9-fold from 3549956 to 134613491 in the case of up to three variable post translational modifications of oxidation (methionine) and phosphorylation (serine, threonine and tyrosine).

Third, with the great progress of liquid chromatography and mass spectrometry, the generation rate of tandem mass spectra is increasing remarkably. A mass spectrometer such as Thermo Electron (Waltham, MA) LTQ generates about five tandem mass spectra per second and Velos generates more than ten tandem mass spectra per second. Although the performance of computing hardware is improving steadily, it cannot catch up with the progress in the generation rate of tandem mass spectra.

To address these problems, some software tools accelerate processing by efficiently organizing the protein sequence database. SEQUEST [1], Crux [7] and pFind [10] use peptide indexing to accelerate tandem mass spectra identification. Tang [12] uses a peptide and b/y ions index. Lu and Chen use a suffix tree for fast tag-based searching[13]. Inspect uses a fast trie-based search for scanning of the database with sequence tags [14]. Edwards and Lippert proposed that elimination of redundant candidate peptides can decrease the identification time because it decreases the corresponding peptide-spectrum matching times. In 2004, Edwards applied a compressed sequencing-by-hybridization graph for sequence database compression for peptide identification [15] and used it for expressed sequence tag searching [16]. This approach compresses the sequence and eliminates most but not all of the peptide redundancy. In 2002, Edwards and Lippert implemented the Simultaneous Linear Scan and Suffix Tree Traversal algorithm for peptide candidate generation[17]. They also proposed the use of the suffix array as a compact representation of a sequence database that can eliminate candidate redundancy. However, there are no details in their paper about how the suffix array is used and how well it performs, and they did not implement it in any search engines.

Based on our daily experience of research and use of search engines, we propose that four aspects are important for choosing a data structure. First, it should improve the identification time efficiency, because that is the aim of using it. Second, it should not require much time and space for construction. Third, it should not affect accuracy. Fourth, it should be flexible to use, as flexibility is important for a search engine. For example, Mascot creates binary formats for ease of input, but it does not perform formal indexing, because a new index would be required for each combination of search parameters [2].

Two approaches to data structure organization should be mentioned. The first is to not use any special data structure. When identification starts, proteins in the database are digested online individually to generate all peptides. Then every peptide is matched with the spectra within the mass tolerance window and a peptide-spectrum matching score is given. This method does not require time and space to construct a data structure beforehand, but it cannot speed up the identification. The second approach is to use a peptide index [18]. Proteins are digested offline and the generated peptides are stored on a disk. For every peptide, its mass, position, length, corresponding proteins and other useful information are recorded. All peptides are sorted and the redundant peptides are eliminated. Any search engine will only need to read the peptide index from the disk to obtain the non-redundant peptides and match them with the spectra. Many search engines implement this data structure to speed up identification, such as SEQUEST [1], Crux [7] and pFind [10]. These search engines use peptide indexing to eliminate redundant peptides and accelerating query response time. The approach of peptide indexing can speed up the identification but it requires much time and space for construction. Furthermore, the time and space requirement can be 100-fold greater for non-specific digestion. In addition, when the search parameters, such as the maximum number of missed cleavage sites, the maximum length or the maximum mass of putative peptides are changed, a new index must be constructed.

The advantages and the disadvantages of these two approaches are obvious. In this study, we propose ABLCP, an algorithm based on the longest common prefix, to organize the database efficiently to retain the advantages and avoid the drawbacks of these approaches.

First, ABLCP sharply improves the identification time. Many peptides appear in more than one protein because there are many homologous proteins in a protein sequence database. The peptide redundancy ratios of some databases are very high. Removal of the redundant peptides can reduce the corresponding peptide-spectrum matching times and thereby decrease the identification time. Compared to an approach that does not use a special data structure, the experiments presented in Results section show that ABLCP can decrease the identification time by about 50%.

Second, compared to the approach using peptide indexing, ABLCP requires less time and space for construction. The construction time for ABLCP is very short: it only needs tens of seconds for normal databases such as IPI-Human and several minutes for large databases such as Uniprot/SwissProt. The additional space needed for ABLCP is equivalent the original database space, which is only half the space needed for full-specific digestion peptide indexing with pFind. The additional time and space for peptide indexing increases remarkably for semi or non-specific digestion, sometimes up to 100-fold over that required for full-specific digestion. However, with ABLCP, the non-specific and full-specific digestions require the same time and space.

Third, ABLCP does not cause any accuracy loss. ABLCP eliminates the redundant candidate peptides by the property of longest common prefix. For database searching, the enzymatic digestion poses a challenge to this algorithm because if the property of longest common prefix is used directly, then some candidate peptides may be omitted. However, some adjustments can be made to this algorithm to ensure no candidate peptides are omitted, thus this algorithm can increase the speed without a loss of accuracy.

Finally, ABLCP is flexible to use. ABLCP uses online digestion, thus it is subject to fewer restrictions. When the digestion parameters are changed, such as the maximum number of missed cleavage sites or the maximum length or mass of putative peptides, the peptide indexing needs to be constructed again, but this is not necessary for ABLCP. ABLCP is more flexible and is only dependent on the database and enzyme.

## Results

In this study, all of the experiments were performed on a Windows XP machine with 4 GB RAM and 2 Intel(R) Xeon(R) CPUs, each of which had 2 1.60 GHz cores. All of the programs were implemented in C/C++ language. Unless otherwise specified, tryptic digestion was assumed, and for site-specific digestion, up to two missed cleavage sites were assumed.

In the METHODS section, we will show how this algorithm ensures that no candidate peptides are omitted and how it performs online digestion. In this section, we evaluate this algorithm through two sets of tests. In the first test, we compare the peptide and protein identification time among the following three workflows: workflow-1, with no special data structure; workflow-2, with peptide indexing; workflow-3, with ABLCP. In the second test, we compare the time and space cost between ABLCP and peptide indexing.

Workflow-1 and workflow-2, implemented in pFind, were compared with workflow-3, using ABLCP. In our previous studies [18], the efficiency of peptide indexing in pFind was compared with SEQUEST, Mascot and X!tandem. pFind requires much less time and space than SEQUEST for construction of the peptide indexing and requires less time than Mascot and X!Tandem for peptide and protein identification. The identification accuracy of pFind, Mascot and SEQUEST was investigated in one of our previous studies [10].

### Identification time

Two experiments were performed that tested two data sets, the ISB data set [19] and mouse liver data set [20], from cited publications. In the ISB data set, all of the ten raw files of the LTQ Data on Mix 1 were chosen to be searched against the 18 proteins merged with the Uniprot/SwissProt V57.9 protein sequence database. In the mouse liver data set, two raw files of pTyr peptides were chosen to be searched against the IPI-Mouse V3.72 protein sequence database [11].

Experiment 1 on the ISB data set used full-specific enzymatic digestion and Experiment 2 on the mouse liver data set used non-specific digestion. The searching parameters are shown in Table 1 and search time is shown in Table 2. The three workflows are used in each group. These experiments show the comparison of the peptide and protein identification time cost.

**Table 1:** The parameters of database searching experiments

	Instrument	LTQ
	Spectra	107666 spectra extracted from ten raw files
Exp1	Database	18 purified proteins with the Uniprot/SwissProt protein sequence database(1020188 protein sequences, target + reversed), total around 460 MB
	Digestion way	Site-specific digestion
	Tolerance	Precursor: +/- 3Da; Fragment: +/- 0.5 Da
	Modifications	Fixed: Carbamidomethylation (C) Variable: Oxidation (M)
	Instrument	LTQ-FT or LTQ-Orbitrap
	Spectra	13816 spectra extracted from two raw files
	Database	IPI-Mouse protein sequence database(113914 protein sequences, target + reversed), total around 66 MB
Exp2	Digestion way	Non-specific digestion
	Tolerance	Precursor: +/- 12 ppm; Fragment: +/- 0.5 Da
	Modifications	Fixed: Carbamidomethylation (C) Variable: Phosphorylation (S, T, Y) Oxidation (M),

**Table 2:** Peptide and protein identification time for the three workflows

Workflow	Experiment 1	Experiment 2
Workflow-1	3679	8278
Workflow-2	2236	4430
Workflow-3	2228	4111

Workflow-1, with no special data structure; workflow-2, with peptide indexing;

workflow-3, with ABLCP. The unit of time is minutes.

From Table 2, in Exp 1, use of peptide index or ABLCP can save around 40% of the identification time versus not use a special data structure, with the time decreasing from 2.5 days to 1.5 days. In Exp 2, the workflow with ABLCP saves around 50% of the identification time, and the workflow with peptide indexing saves around 46% of the identification time. The time is reduced from 5.7 days to 2.9 days with ABLCP and to 3.1 days with peptide indexing. The workflow with peptide indexing is not as efficient as workflow with ABLCP, because peptide indexing spends much time on reading the indexing files.

These two approaches eliminate redundant candidate peptides in protein sequence databases and reduce the corresponding peptide-spectrum matching times, thereby decreasing the identification time. The redundancy ratio of the two databases is shown in Table 3. For these two large databases, one has a redundancy ratio around 55% and the other around 33%. Removal of the redundant candidate peptides can decrease the identification time in these large databases.

**Table 3:** The peptide redundancy ratio of two protein sequence databases

Database	Peptide Number	Full-specific	Semi-specific	Non-specific
	Non-redundant	3549956	55908454	626871441
Human	Redundant	8022636	128308391	1401160777
	Redundancy	55.7%	56.4%	55.2%

	Non-redundant	24915278	395305609	4525189544
SwissProt	Redundant	37646081	601652577	6554527058
	Redundancy	33.8%	34.2%	31.0%

The peptide redundancy ratio of the IPI-Human V3.65 and Uniprot/SwissProt V56.2 protein sequence databases. The length of the peptides is limited from 6 to 60 amino acids.

Workflow-1 is slow but without additional storage space or time cost for construction. Both workflow-2 and workflow-3 can speed up the identification, but have some additional storage space and time cost. Because reduction of the cost is important for identification efficiency, we should compare the storage space and time cost to choose a better data structure for identification.

### Storage space and time cost for construction

Three aspects are evaluated to compare the cost between ABLCP and peptide indexing. The first is the additional storage space needed for the data structure. The second is the time needed to construct the ABLCP or peptide indexing. The third is the time needed for identification using the ABLCP or peptide indexing. These aspects were tested in the IPI-Human V3.65 and Uniprot/SwissProt V56.2 protein sequence databases for full-, semi- and non-specific digestion. The peptide length range is from 6 to 60 amino acids.

The first aspect that we compared is the additional storage space for the data structure. Table 4 shows the storage space needed for ABLCP and peptide indexing in two databases for full-, semi- and non-specific digestion. For peptide indexing, it is necessary to record information such as the mass, the position, the length and other information of every candidate peptide. For ABLCP, because the length of a peptide is limited and each peptide usually contains no more than 100 amino acids, implying the length of a string representing a peptide sequence is not longer than 100, only one byte is needed for each LCP. Table 4 shows that peptide indexing needs more storage space than ABLCP, particularly with non-specific digestion. For full-specific digestion, ABLCP requires only half of the additional storage space of peptide indexing. For non-specific digestion, ABLCP requires the same additional storage space as site-specific digestion, but this additional storage space increases significantly (up to 100-fold) for peptide indexing. In the Uniprot/SwissProt V56.2 database, peptide indexing requires 424 MB for site-specific digestion and 65122 MB for non-specific digestion. Table 4 shows that the additional storage space required for ABLCP is only about 1/320 of that of peptide indexing for the IPI-Human database and about 1/470 of that of peptide indexing for the Uniprot/SwissProt database. Non-specific digestion could be used to match peptides when the enzyme specificity is unknown, such as with endogenous peptides.

**Table 4:** The additional storage space needed for ABLCP and peptide indexing

Database	Workflow	Full-specific	Semi-specific	Non-specific
Human	ABLCP	30	60	30
	Peptide Index	67	939	9799

SwissProt	ABLCP	137	274	137
	Peptide Index	424	6081	65122

The experiments were performed on the IPI-Human V3.65 and Uniprot/SwissProt V56.2 protein sequence databases for full-, semi- and non-specific digestion. The unit of storage space is MB.

The second aspect that we compared is the time needed to construct ABLCP or peptide indexing. Table 5 shows the time needed to construct ABLCP and peptide indexing for two databases for full-, semi- and non-specific digestion. In the large database, the non-specific digestion costs several hours for peptide indexing, but the time spent for ABLCP is no more than several minutes, and the data show that ABLCP is 100 times quicker than the peptide indexing.

**Table 5:** The time needed to construct ABLCP and peptide indexing

Database	Workflow	Full-specific	Semi-specific	Non-specific
Human	ABLCP	41	41	41
	Peptide Index	50	603	6475

SwissProt	ABLCP	196	196	196
	Peptide Index	242	2919	24828

The experiments were performed on the IPI-Human V3.65 and Uniprot/SwissProt V56.2 protein sequence databases for full-, semi- and non-specific digestion. The unit of time is seconds.

The third aspect that we compared is the time required to use ABLCP or peptide indexing for identification. For peptide indexing, this time is spent on reading the index and sequence files from a disk. For ABLCP, the time is spent on reading files from a disk and online digestion. Reduction of this time can reduce the identification time and improve the analysis efficiency. Sometimes, especially for high-accuracy instruments, peptide indexing can skip some peptides with various meta-data, sequence, or mass properties, so this time may be short for peptide index. For most other situations, this time is proportional to the peptide number. Table 6 shows the time required to use ABLCP and peptide indexing for identification in two databases for full-, semi- and non-specific digestion. The data show that ABLCP is much quicker than the peptide indexing and that the time required is less than 20% of peptide indexing on average.

**Table 6:** The time needed to read peptides from the disk

Database	Workflow	Full-specific	Semi-specific	Non-specific
Human	ABLCP	3	108	144
	Peptide Index	20	317	3588

SwissProt	ABLCP	16	441	1032
	Peptide Index	144	2301	25283

The time needed to read peptides from the disk for peptide indexing or online digestion for ABLCP. The experiments were performed on the IPI-Human V3.65 and Uniprot/SwissProt V56.2 protein sequence databases for full-, semi- and non-specific digestion. The unit of time is seconds.

These tables show that the construction time and query time required to use special data structures is low, especially for ABLCP, which requires no more than several minutes. The additional storage space needed for ABLCP is small. However, these data structures can decrease the identification time remarkably, as shown in Table 2. Thus, it is worthwhile to use these data structures. In terms of storage space, construction time and query time, ABLCP is much more efficient than peptide indexing, especially for large databases and non-specific digestion. The cost for peptide indexing in a large database with non-specific digestion is barely acceptable for a normal personal computer, because this cost can even be tens of GB and several hours. But this cost is not a problem for ABLCP, as the storage space and time cost for ABLCP is much less than that of peptide indexing.

## Discussion

ABLCP is lossless and independent of scoring model, so it can also be designed to be used in other search engines. In the future, we intend to transplant our algorithm to a parallel environment for searching. Searching in parallel is another way to speed up identification. For ABLCP, because every character and LCP position is independent after construction, it is only necessary to divide the protein coupled with LCP into several parts for searching in parallel.

## Conclusions

This paper explores an algorithm called ABLCP that organizes protein databases efficiently. This algorithm eliminates redundant candidate peptides in protein sequence databases and reduces the time of peptide-spectrum matching, thereby decreasing the identification time. We compare ABLCP with two other workflows: workflow-1, with no special data structure, and workflow-2, with peptide indexing. Compared to workflow-1, ABLCP can decrease identification time by about 50%. Compared to workflow-2, ABLCP is proved to be more efficient in terms of time and storage space required. This algorithm is based on the property of LCP with some adjustments made for site-specific digestion. In addition, ABLCP is more flexible. ABLCP uses online digestion, and thus, it is subject to fewer restrictions.

## Methods

### Basic Notion

#### Basic notions

A string *T *= *T *[0*...n*) =*t_0_t_1_...t_n-1 _*is the input to the suffix array

construction algorithm. In this paper, the input string *T *is the connection of all of the protein sequences represented by strings in FASTA database files, and a character '$' is appended to each protein sequence string to separate them.

For *i *∈[0, *n*), *Suffix*[*i*] denotes *T *[*i*, *n*) = *t_i_t_i+1_...t_n-1_*. An array *SA*[0...n) denotes the ranked suffixes, *SA*[*j*] = *i *if and only if *Suffix*[*i*] is the *j*th suffix of *T *in the ascending lexicographical order. Another array *Rank *[*0...n*) is used for the inverse *SA*. *Rank*[*i*] = *j *if and only if *SA*[*j*] = *i*, and it means that *Suffix*[*i*] is ranked *j*th in the ascending lexicographical order of all suffixes.

**Figure 1:**
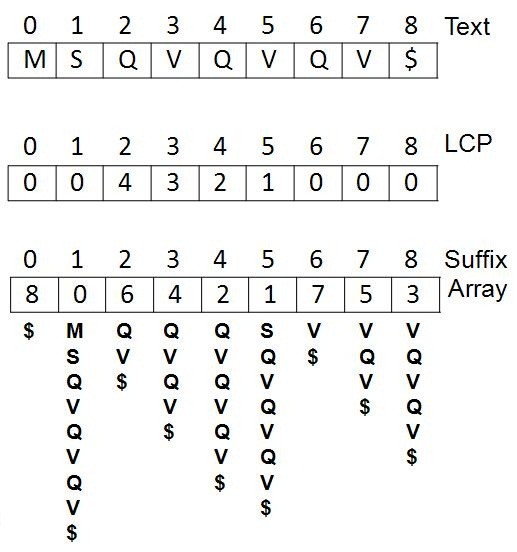
**An example of the corresponding suffix array and LCP for an input text string**. The first row is the input text string *T *= *T *[0*...n*) = MSQVQVQV$. *n *is 8 and the index begins with 0. The second and third rows are the corresponding LCP and suffix array. Take the value at index 2 to explain the meaning. *SA*[*2*] is 6, which means that the third suffix in the ascending lexicographical order is the *Suffix*[*6*] and this suffix is "QV$". *LCP*[*2*] is 4, which means that the longest common prefix between the *Suffix*[*2*] "QVQVQV$" and its previous suffix (in the lexicographical order, *Suffix*[*4*]"QVQV$") is 4.

LCP denotes the longest common prefix of two adjacent suffixes, which are in the ascending lexicographical order. For *i *∈[*0*, *n*), *LCP *[*i*] denotes the length of the longest common prefix of adjacent suffixes *Suffix*[ *SA*[*Rank*[*i*]-1]] and *Suffix*[*i*]. Define *lcp *(*y*, *z*) as the length of longest common prefix of strings *y *and *z*, *LCP*[*i*] = *lcp*(*T*[*SA*[*Rank*[*i*]-1]...*n*), *T*[*i*...*n*)). Define *LCP*[*SA *0] to be zero. An example of the corresponding suffix array and LCP for an input text string is shown in Figure 1.

#### Suffix array

As surveyed by Puglisi et al in 2007 [21], the suffix array was proposed in 1990 [22]. This algorithm is used as an alternative to the suffix tree [23] but with higher memory space efficiency. When the suffix array was first proposed, the prefix-doubling construction algorithm was used with time complexity O(*n*log*n*), where *n *is the size of the text, such as in the algorithms by Manber and Myers(MM) [22] and by Larsson and Sadakane(LS) [24]. Since then, many other types of algorithms have been proposed, such as recursive algorithms, induced algorithms and hybrid algorithms. Some of them have O(*n*) time complexity, such as the algorithm DC3 [25]. Other algorithms have higher time complexity in the worst case but they perform well in practice, such as the induced algorithms by Manzini and Ferragina(MF) [26] and by Maniscalco and Puglisi(MP) [27]. Furthermore, these algorithms require less memory. For suffix array construction algorithms, minimal asymptotic time complexity is preferable, and low practical time cost and high memory space efficiency are crucial. In this paper, we chose four different types of suffix array construction algorithms, i.e., LS [24], DC3 [25], MF [26] and MP [27] to test their performance in protein sequence databases. Because the alphabet of protein databases is small (just twenty characters), we needed to determine which algorithm was most suitable. The program of LS was implemented by our group and the other programs were downloaded from the respective papers or from the websites listed in the papers. Table 7 shows the construction time of the four construction algorithms. We choose the MP [27] algorithm for implementation in the search engine pFind for further experiments because it was the quickest among the four algorithms in protein sequence databases and its memory space efficiency is sufficient. The main idea of the MP algorithm is to break the suffixes into groups, assigning ranks to the suffixes in each group in lexicographical order and then using these ranks to subsequently speed up the assignment of ranks to other suffixes. When the algorithm completes, every suffix has been assigned a unique lexicographic rank, enabling the suffix array to be computed.

**Table 7:** The construction time of the four algorithms for the two databases

	IPI-Human	Uniprot/SwissProt
Prefix-doubling algorithm LS	290.7	--
Recursive algorithm DC3	109.6	--
Induced algorithm MF	24.3	111.5
Induced algorithm MP	17.9	92.0

The table does not have the time of algorithms LS and DC3 for the Uniprot/SwissProt V56.2 database, because the two algorithms required too much memory space and could not be constructed in memory. The unit of time is seconds.

#### LCP

The most important concept used in this paper is the LCP. The LCP guarantees that there are no redundant candidate peptides at the time of online generation. The LCP is induced from the suffix array. The first O(n) time complexity LCP construction algorithm was proposed by Kasai et al in 2001 [28]. This algorithm is simple and efficient. Many other algorithms with higher memory space efficiency were subsequently proposed. For example, Puglisi proposed a space-time tradeoff algorithm in 2008 [29]. In this paper, we implement Kasai's algorithm to induce the LCP.

The LCP guarantees that there are no redundant candidate peptides. However, for database searching, the enzymatic digestion poses a challenge to this algorithm. If the LCP is used directly, then some candidate peptides may be omitted. Thus, we make some adjustments to the LCP after it is constructed to accommodate the enzymatic digestion.

### Identification Workflow

**Figure 2:**
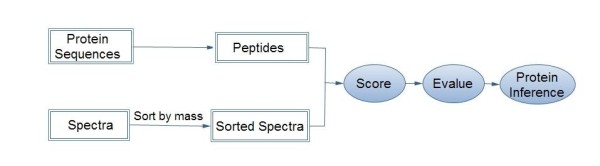
**Shared processes of the three workflows**. Spectra are sorted by their precursor masses and the candidate peptides are obtained from a protein sequence database. Then all of the spectra within the specified mass tolerance window are found for the candidate peptide, and each peptide-spectrum match is scored. Evaluation and protein inference occur at the end of the matching and scoring stage.

In this study, we choose two approaches to compare to ABLCP, resulting in the following three workflows: workflow-1, with no special data structure; workflow-2, with peptide indexing; and workflow-3, with ABLCP. We implement these three workflows in pFind to compare their efficiency. Most processes of the three workflows are the same. These processes are shown in Figure 2.

The difference among the three workflows is in the step that converts protein sequences to peptides. In workflow-1, with no special data structure, protein sequences in the database are digested online one by one to generate all peptides. In workflow-2, with peptide indexing, proteins are digested offline and the generated peptides are stored on disk. For every peptide, its mass, position, length, corresponding proteins and other useful information are recorded. All peptides are sorted and the redundant peptides are eliminated. For any search engine, it is only necessary to read the peptide index from the disk to obtain the non-redundant peptides and match them with spectra. In workflow-3, with ABLCP, in the process of preprocessing, the suffix array is constructed by the MP algorithm [27], and the associated array LCP is constructed by Kasai's algorithm [28]. After that, some adjustments are made to allow LCP to accommodate the enzyme. Then the LCP is restored on disk and the suffix array is discarded. In the process of identification, the protein sequence database and the LCP are loaded from disk to memory then digested online. All three workflows transform the protein sequence database into a binary form for quick block reading before the step that converts protein sequences to peptides.

In the following section, we will demonstrate how to digest the proteins online with ABLCP and prove that this online protein digestion with ABLCP can completely eliminate redundant candidate peptides without any loss of accuracy. The non- and site-specific digestions are discussed separately. Finally, we will show how to speed up the online protein digestion and how to perform protein inference.

### ABLCP workflow

#### Non-specific digestion

The protein sequences in the databases are expressed as strings, so the problem of non-specific digestion can be considered as the problem of obtaining all substrings from the original string. The pseudo code of generating all non-redundant substrings from an original string with ABLCP is illustrated in Algorithm 1: GetAllSubStrings.

** Algorithm 1**: GetAllSubStrings--generating all substrings from the original string

**   Input**: The original string *T *, the length of *T *is n, the array of *LCP*

**   Output**: all the substrings *subStrings*

**      For ***i *= 0: (*n *- 1)

**         For ***length *= (*LCP*[*i*] + 1): (*n *- *i*)

               *subSrings*.push_back(*T*[*i*, *i *+ *length*))

         **End**

      **End**

**Figure 3:**
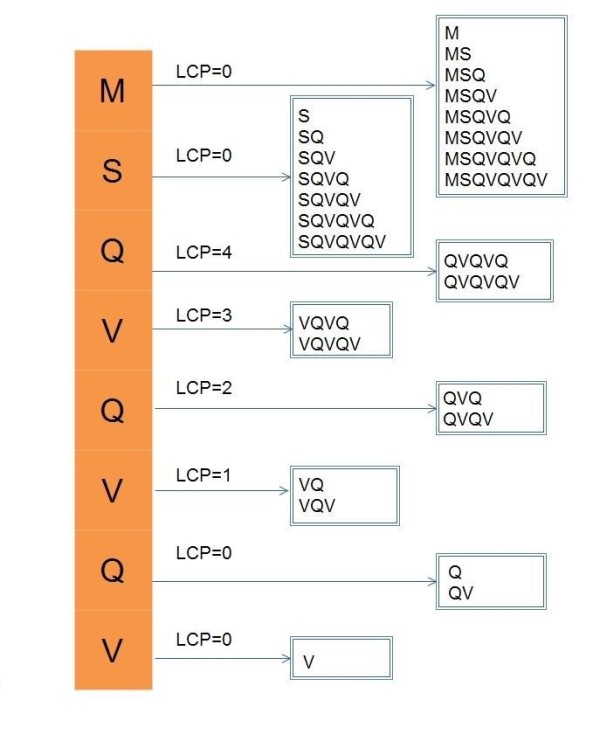
**An example of the algorithm GetAllSubStrings**. The original string T is {MSQVQVQV$}, and the LCP is {0, 0, 4, 3, 2, 1, 0, 0, 0}. Because the '$' does not belong to the protein sequence database, so the '$' is omitted from in the For loop. Take the suffix "VQVQV" as an example. The corresponding LCP is 3 and this suffix generates substrings at length from 4 (LCP plus one), so this suffix generates two substrings "VQVQ" and "VQVQV".

   One example (the string in Figure 1) of the algorithm GetAllSubStrings is shown in Figure 3.

In the algorithm GetAllSubStrings, for every *i *∈[0, *n*), *Suffix*[*i*] generates all of its prefixes from the length of (*LCP*[*i*]+ 1) to the length of this suffix, so Property 1 and Property 2 can be concluded:

**Property 1**. In the algorithm GetAllSubStrings, if and only if substring *S *is prefix of *Suffix*[*i*] and the length of *S *is larger than *LCP*[*i*], *S *can be generated by *Suffix*[*i*].

Property 2

If substring *S *can be generated by *Suffix*[*i*], which means that *LCP*[*i*] is smaller than the length of *S*, then *Suffix*[*SA*[*Rank*[*i*] - 1]] will not contain *S *as prefix.

All non-redundant substrings have two meanings. The first is that all of the substrings can be obtained, and the second is that no two obtained substrings are the same. We need to prove the following two theorems to prove that the algorithm GetAllSubStrings can obtain all non-redundant substrings:

**Theorem 1**. All of the substrings can be obtained.

**Theorem 2**. No two obtained substrings are the same.

With Property 1 and Property 2, these two theorems can be proved. The proofs are shown in Additional file 1. With these two theorems, it is proven that the algorithm GetAllSubStrings generates all non-redundant substrings.

In practice, some details should be added to the algorithm GetAllSubStrings. For example, the length and the mass of the peptides are bounded within a small range due to the limited detection capability of mass spectrometry. In addition, in search engines, it is not realistically possible to obtain all of the substrings at one time in the For loop, so the program needs to hold the temporary variables and obtain substrings one by one.

#### Site-specific digestion

For site-specific enzymatic digestion, it is slightly more complex to generate all non-redundant peptides using the algorithm GetAllSubStrings, because there are some restrictions at both ends of a peptide. Take C-terminal full-specific tryptic digestion as an example, and the N-terminal digestion is the reverse situation of the C-terminal. A substring is a legal peptide only if its previous character is 'R' or 'K', or if it is at the N-terminal of a protein. We define the type of suffixes that can generate legal peptides as SS (Specific Suffixes, the suffix whose previous character is 'R' or 'K' or is at the N-terminal of a protein). Because a substring is a legal peptide only if it is the prefix of a suffix in SS, so Property 1 does not comply with the site-specific digestion and the algorithm GetAllSubStrings cannot generate all non-redundant site-specific digestion peptides. One example is shown in the Additional file 2.

With Property 1 and Property 2, the algorithm GetAllSubStrings can generate all non-redundant substrings for non-specific digestion. If Property 1 and Property 2 comply with the site-specific digestion, then the algorithm GetAllSubStrings can also generate all non-redundant substrings for site-specific digestion. However, for site-specific digestion, two problems arise. First, only the suffixes in SS can generate legal peptides, whereas others cannot (under the site-specific digestion rule), indicating that not every substring *S*, which is the prefix of one suffix *Suffix*[*i*] and whose length is larger than *LCP*[*i*], is a legal peptide. Second, if the first occurrence of a peptide is in a suffix which is not in SS, the algorithm GetAllSubStrings will never consider this peptide again in a suffix which is in SS. However, it actually needs to. This indicates that even the length of substring *S *is not larger than *LCP*[*i*], substring *S *may need to be generated by *Suffix*[*i*].

However, two strategies can be proposed to deal with these problems separately. First, we only consider the suffixes in SS and discard others. Then, a substring *S*, which is a prefix of one suffix *Suffix*[*i*] and whose length is larger than *LCP*[*i*], is a legal peptide. Second, we adjust the LCP to the value between adjacent suffixes in SS. Then, the suffixes not in SS will not affect the suffixes in SS. Thus, the algorithm GetAllSubStrings will consider the peptide whose first occurrence is in a suffix which is not in SS, and *Suffix*[*i*] only needs to generate substrings whose length is larger than *LCP*[*i*].

After application of the two strategies, if and only if substring *S *is the prefix of *Suffix*[*i*] and the length of *S *is greater than *LCP*[*i*], *Suffix*[*i*] needs to generate substring *S*. Thus, Property 1 complies with the site-specific digestion. If only the suffixes in SS are considered, then Property 2 can also be induced by adjusting the LCP.

The implementation of sole considering the suffixes in SS with discarding of the others is straightforward. When scanning a suffix, if the suffix's previous character is not 'R' or 'K', then discard this suffix. This means that in the first For loop of the algorithm GetAllSubStrings, if *T*[*i *-1] is not 'R' or 'K', then the first For loop can skip to the next suffix immediately.

The LCP of two suffixes is the minimum of the LCP of all pairs of adjacent suffixes between them, which was proven in a previous paper [22]. With the variable in the section of Basic notions, that is,

(1) $$lcp\left(Suffix\left[SA[x]\right],Suffix\left[SA[z]\right]\right)=\underset{x<y\leq z}{\min}\left\{lcp(Suffix\left[SA[y-1]\right],Suffix\left[SA[y]\right]\right\}$$

so that steps of the adjustment of LCP can be illustrated in Algorithm 2: AdjustLCP.

** Algorithm 2**: AdjustLCP -The adjustment of LCP for site-specific digestion

**   Input: **The original string *T *, the length of *T *is *n*, the array of *LCP*, *SA*

**   Output: **The adjusted array *LCP*

**      For **(*i *= 0; *i *<*n *; ++*i*)

      {

         **If ***Suffix*[*SA*[*i*]] is in SS

            {

               **For**(*k *= *i *-1; *k *> 0; -- *k *)

               {

                  **If ***Suffix*[*SA*[*k*]] is in SS

                     **break**

                  **Else**

                  {

                        **If ***LCP*[*SA*[*k*]] <*LCP*[*SA*[*i*]]

                           *LCP*[*SA*[*i*]] = *LCP*[*SA*[*k*]]

               }

            }

         }

   }

After the two strategies are implemented, the algorithm GetAllSubStrings can generate all non-redundant site-specific digestion. But some other situations exist. Take trypsin/p (C-terminus of 'K/R', unless followed by 'P') as an example there are some other restrictions for digestion besides the restrictions for trypsin cleavage: a character 'KR' followed by 'P' cannot be a cleavage site. Even after the adjustment of LCP by algorithm AdjustLCP, some peptides may be ignored. The adjustment of LCP for trypsin/p digestion and its proof is shown in the Additional file 3.

After the adjustment of LCP, retain a substring in the second For loop of the algorithm GetAllSubStrings if the character *T*[ *i *+ *length *- 1] is 'R' or 'K' and discard it if not. Additionally, the missed cleavage sites for site-specific digestion should also be considered. The pseudo code for generation of all non-redundant substrings for site-specific digestion is illustrated in Algorithm 3: GetAllSpecificSubStrings.

** Algorithm 3**: GetAllSpecificSubStrings--generating all site-specific substrings from the original string

**   Input**: The original string *T *, the length of *T *is n, the array of *LCP *adjusted by the algorithm AdjustLCP

**   Output**: all the site-specific substrings *subStrings*

**      For ***i *= 0: (*n *- 1)

      {

         **If ***T*[ *i *- 1] is not 'R' or 'K'

            **continue**

**         For ***length *= (*LCP*[*i*] + 1): (*n *- *i*)

         {

            **If ***T*[ *i *+ *length *- 1] is not 'R' or 'K'

               **continue**

            **Else**

            {

               ++MissCleavageSitesNum

               **If **MissCleavageSitesNum > MaxMissCleavageSitesNum

                  **break**

               *subSrings*.push_back(*T*[*i*, *i *+ *length*))

            }

         **}**

      **}**

#### Semi-specific digestion

For semi-specific digestion, all of the non-redundant semi-specific digestion peptides can be obtained by two parts: The first part consists of generation of peptides by the algorithm GetAllSubStrings with LCP not adjusted, but a peptide is retained only when its first right amino acid is a cleavage site. The second part consists of generation of peptides by the algorithm GetAllSpecificSubStrings with LCP adjusted, but a peptide is retained only when its first right amino acid is not a cleavage site. The proof that ABLCP can obtain all non-redundant semi-specific digestion peptides is shown in the Additional file 4.

#### Speeding up online digestion

To speed up the online protein digestion, we optimized the implementation. Data are read from the disk by blocks. The mass value of every amino acid is multiplied by 10000 for storage and computation in integer form but still with sufficient accuracy retained. Additionally, duplicate calculations are removed. The peptides always have some overlap because there are some missed cleavage sites. We record the mass from the beginning of a protein up to each amino acid and obtain every peptide mass by deducting the mass from the end position of the peptide to the beginning. For site-specific digestion, we record every potential cleavage sites and how many missed cleavage sites there are from the beginning of the protein, so that we can quickly locate the potential cleavage sites. With these optimizations, the online digestion is very quick. Experiments show that the time required to read files from the disk and perform online full-specific enzymatic digestion is only 3 seconds for the IPI-Human V3.65 protein sequence database and 16 seconds for the Uniprot/SwissProt V56.2 database.

#### Protein inference

At the end of peptide identification of tandem mass spectra, the protein sequence, accession number and description corresponding to each peptide should be determined. The Aho-Corasick algorithm [30], which has been mentioned and implemented for scanning a database with sequence tags in the search engine InsPecT [14], is chosen to solve this problem. With a linear pre-processing time, the Aho-Corasick algorithm can construct all the identified peptides to a trie-automaton. Then a single pass through the database can handle all of the identified peptides. The protein sequence is scanned against the trie-automaton one by one, and the identified peptides that are contained by this protein are recorded. The accession number and description corresponding to the protein sequence can also be recorded for the identified peptides in this step. This step is very fast. An experiment tested on 40000 identified spectra with the IPI-Human database (target + reversed) took less than 20 seconds.

## Authors' contributions

CZ, HC and SH designed this study. CZ implemented this algorithm and performed the experiment. CZ, LW, YL and YW implemented this algorithm in software. CZ, YF and RS analyzed the data. All authors have read and approved the final manuscript.

## Availability and Requirements

Project name: ABLCP project;

Project home page: http://pfind.ict.ac.cn/pfind2dot5/index.htm;

Operating system(s): Platform independent;

Programming language: GCC 3.4.5 or higher;

Licence: Please read our licence at http://pfind.ict.ac.cn/files/License.pdf;

Any restrictions to use by non-academics: Licence needed.

## Supplementary Material

Additional file 1 **The proof of Theorem 1 and Theorem 2**. Click here for file

Additional file 2 **The example that Property 1 does not comply with site-specific digestion**. Click here for file

Additional file 3 **The adjustment of LCP for trypsin/p cleavage and its proof**. Click here for file

Additional file 4 **The proof that ABLCP can obtain all non-redundant semi-specific digestion peptides**. Click here for file
