# Supplementary material for: Speeding up tandem mass spectrometry-based database searching by longest common prefix
Source: BMC Bioinformatics. 2010 Nov 25;11:577. doi: 10.1186/1471-2105-11-577 (PMC3000425; doi:10.1186/1471-2105-11-577)
Supplement: Additional file 1 — The proof of Theorem 1 and Theorem 2. [file 1471-2105-11-577-S1.PDF]

Theorem 1. All of the substrings can be obtained.

Proof. We know that all of the substrings are the prefix of some suffixes. For any substring *S*, suppose that *Suffix*[*i*] is the smallest suffix of all the suffixes which have the prefix of *S*. *Suffix*[*SA*[*Rank*[*i*] – 1]] is smaller than *Suffix*[*i*] so it does not contain *S* as a prefix. In addition, *LCP*[*i*] must be smaller than the length of *S*. As a result, with the situation that substring *S* is a prefix of *Suffix*[*i*] and the length of *S* is larger than *LCP*[*i*], substring *S* can be generated by *Suffix*[*i*] by Property 1. End of proof.

Theorem 2. No two obtained substrings are the same.

Proof. Let *i* and *k* be the positions of the two copies of the repeated substring *S*, and assume that *Rank*[*i*] < *Rank*[*k*]. If *Rank*[*i*] + 1 == *Rank*[*k*] then property 2 is violated. Otherwise， all suffixes *j* with *Rank*[*j*] between R*ank*[*i*] and *Rank*[*k*] must have prefix *S*, and hence *lcp*(*Suffix*[*SA*[*Rank*[*k*]-1]],*Suffix*[*k*]) >= length(*S*), violating property 2. End of proof.
