# Supplementary material for: Speeding up tandem mass spectrometry-based database searching by longest common prefix
Source: BMC Bioinformatics. 2010 Nov 25;11:577. doi: 10.1186/1471-2105-11-577 (PMC3000425; doi:10.1186/1471-2105-11-577)
Supplement: Additional file 2 — The example that Property 1 does not comply with site-specific digestion. [file 1471-2105-11-577-S2.PDF]

The example that Property 1 does not comply with site-specific digestion.

Take full-specific tryptic digestion as example, substrings are legal only if their first left character is ‘R’ or ‘K’, or they are begin at the beginning of the proteins. We define this kind of suffixes as SS (specific suffixes，the suffix which previous character is ‘R’ or ‘K’, or it is at the N-terminal of a protein). If the protein sequence *T* = KASTYRLASTYRC is full-specific enzymatic digested by tryptic enzyme and the maximum missed cleavage sites is zero, one peptide sequence ASTYR should be obtained but actually not in the algorithm GetAllSubStrings. The reason is as follow. For suffix ASTYRC, its left character is ‘L’ and it is not in SS, so it can’t generate any substring. For suffix ASTYRLASTYRC, it follows the suffix ASTYRC in ascending lexicographical order and *LCP*[1] is five(*lcp*(ASTYRC, ASTYRLASTYRC)), the length of peptide sequence ASTYR is five so it can’t be generated by Property 1. Furthermore, no other suffixes can generate the peptide sequence ASTYR, so the peptide is ignored in function the algorithm GetAllSubStrings. This concludes that the algorithm GetAllSubStrings need some adjustment for site-specific digestion.
