# Supplementary material for: Speeding up tandem mass spectrometry-based database searching by longest common prefix
Source: BMC Bioinformatics. 2010 Nov 25;11:577. doi: 10.1186/1471-2105-11-577 (PMC3000425; doi:10.1186/1471-2105-11-577)
Supplement: Additional file 3 — The adjustment of LCP for trypsin/p cleavage and its proof. [file 1471-2105-11-577-S3.PDF]

Trypsin/p site-specific cleavage.

For this LCP based algorithm, some peptides may be ignored, because these two situations may occur: For some suffixes, they can generate a peptide, but this peptide may be discarded for the specific digestion rule; For other suffixes, they are in accordance with the specific digestion rule, but their LCP is too large, so some peptides will not be generated. If the two situations occur for the same peptide, this peptide may be ignored. So it needs to do some small adjustment for LCP to make sure that no peptides are ignored or redundancy.

For trypsin/p (C-terminus of ‘K/R’, unless followed by ‘P’), there are some other restrictions for digestion besides the restrictions for normal trypsin cleavage: a character ‘K/R’ followed by ‘P’ cannot be a cleavage site. If the first character of a peptide is ‘P’, discard this peptide. But if ‘P’ is after a peptide, those two situations may both occur and some peptides may be ignored.

Suppose that the LCP had been adjusted for full-specific trypsin digestion by algorithm AdjustLCP, only the SS (Specific Suffixes, the suffix whose previous character is ‘R’ or ‘K’, or is at the N-terminal of a protein) are taken into consideration, the character ‘X’ indicates any amino acid character and the character ‘#’ indicates any amino acid character except ‘P’. If the following two suffixes both occur, a peptide may be ignored.

XX…XRPXXX…

XX…XR#XXX…

(Suppose that there are h-1 characters ‘X’ before ‘R’ and these prefixes are the same in these two suffixes, where h is any integer larger than one, and no matter how many and what characters ‘X’ after ‘P’ or ‘#’).

Suppose that ‘#’ is larger than ‘P’. The suffix “XX…XRPXXX…” can generate substring “XX…XR”, but “XX…XR” will be discarded for the followed character ‘P’. The suffix “XX…XR#XXX…” will not generate “XX…XR”, because its LCP is h and it will generate peptides from the length of (h + 1), so “XX…XR” will never be generated.

An adjustment of LCP is proposed to resolve this problem when those two situations both occur for the trypin/p enzyme. The details are in algorithm 4: AdjustLCP2 in this additional file. This algorithm is after the algorithm AdjustLCP for normal trypsin cleavage. The main idea of this algorithm is:

For every *Suffix*[*SA*[*i*]], if (*T*[*SA*[*i*]+*LCP*[*SA*[*i*]]- 1] == K/R && *T*[*SA*[*i*] + *LCP*[*SA*[*i*]]] != ‘P’) is true, *LCP*[*SA*[*i*]] may need to be adjusted. Suppose that k is the biggest one which satisfies that k is less than i and *LCP*[*SA*[*k*]] is less than *LCP*[*SA*[*i*]]. If (*T*[*SA*[*k*]+*LCP*[*SA*[*k*]] - 1] == K/R && *T*[*SA*[*k*] + *LCP*[*SA*[*k*]]] ==‘P’) is true, let *LCP*[*SA*[*i*]] minus one.

Algorithm 4: AdjustLCP2 -The adjustment of LCP for Trypsin/p digestion

```
Input:    The original string T , the length of T is n, the array of LCP, SA
Output:   The adjusted array LCP
 For (i = 0; i < n ; ++i)
 {
   If   Suffix[SA[i]] is not in SS
      continue
   If   T[SA[i]+LCP[SA[i]] - 1] == K/R && T[SA[i] + LCP[SA[i] ] ] != P
   {
      For( k = i -1; k > 0; -- k )
      {
         If   Suffix[SA[k]] is not in SS
            continue
         Else If LCP[SA[k]] < LCP[SA[i]]
         {
            If   T[SA[k]+LCP[SA[k]] - 1] == K/R && T[SA[k] + LCP[SA[k]]] == P
                 LCP[SA[i]] = LCP[SA[i]] - 1
            break
         }
      }
   }
 }
```

This algorithm can make sure that all non-redundant substrings affected by the character 'P' can be obtained. The proof is in the following. In the proof, the larger or less relation between suffixes is in the ascending lexicographical order. The suffixes followed by the same number express that they are the same suffix.

First, all of the substrings affected by the character 'P' can be obtained.

For these two kind suffixes "XX…XRPXXX…" and "XX…XR#XXX…", if it exists that some "XX…XR#XXX…" is less than "XX…XRPXXX…", there must be one suffix "XX…XR#XXX…"(1) whose LCP is less than h, so "XX…XR#XXX…"(1) will generate "XX…XR". If all suffixes "XX…XR#XXX…" are larger than "XX…XRPXXX…", there must be one suffix "XX…XRPXXX…"(2), whose LCP is less than h, and one suffix "XX…XR#XXX…"(3), whose LCP is equal to h. The suffixes between these two suffixes are in the form of "XX…XRXXXX…" and those LCP are equal or larger than h. This suffix "XX…XR#XXX…"(3) will find the suffix "XX…XRPXXX…"(2) in algorithm AdjustLCP2, then its LCP will minus one to (h - 1), so this suffix "XX…XR#XXX…"(3) will generate "XX…XR". So no substrings are ignored.

Second, no two obtained substrings affected by the character 'P' are the same.

Once one suffix "XX…XR#XXX…"(4) can generate "XX…XR", its LCP value must be less than h, and the LCP of the following suffixes "XX…XR#XXX…" is equal to or larger than h. For any suffix "XX…XR#XXX…"(5) whose LCP is equal to h, the algorithm

AdjustLCP2 will find the suffix "XX…XR#XXX…"(4), and judge that (*T*[*SA*[*k*]+*LCP*[*SA*[*k*]] - 1] == K/R && *T*[*SA*[*k*] + *LCP*[*SA*[*k*]]] == 'P') is false, so the LCP of the suffix "XX…XR#XXX…"(5) will not be adjusted and the suffix "XX…XR#XXX…"(5) will not generate "XX…XR". As a result, no other suffixes can generate "XX…XR". So no redundant peptides exist.

As a result, all substrings affected by the character 'P' can be obtained and no redundancy. If the restriction is not only 'P' but a character set, just change the 'P' to a character set in the algorithm and proof.

For this algorithm AdjustLCP2, before the second For loop, there is a judgment that (*T*[*SA*[*i*]+*LCP*[*SA*[*i*]] - 1] == K/R && *T*[*SA*[*i*] + *LCP*[*SA*[*i*]]] != P), so this algorithm is quick in practice. However, the time complexity is O(n^2) in worst case, such as for a string "KK….KKQ". To avoid the worst case, we describe an algorithm which lets this time complexity is O(*n*) in worst case, by finding the biggest *k* in O(1) time complexity to discard the second For loop, where *k* is less than *i* and lets *LCP*[*SA*[*k*]] is less than *LCP*[*SA*[*i*]]. This algorithm is as following:

We use a stack to save the index *k* values, and only the indexes of SS (special suffix) are considered. In initial, the stack is empty. When a new *Suffix*[*SA*[*i*]] in the first For loop is coming, it needs to find that biggest *k*. If the top one of the stack satisfies (*LCP*[*SA*[*k*]] < *LCP*[*SA*[*i*]]), this one is the *k* we need. If not, just pop out the top one, until find one that satisfies (*LCP*[*SA*[*k*]] < *LCP*[*SA*[*i*]]) or until the stack is empty. After the period of finding and adjustment, push back the index *i* to the stack.

For every *Suffix*[*SA*[*i*]], it only needs to push back and pop out the index *i* one time, so the time complexity of algorithm AdjustLCP2 is O(*n*). In the stack, the corresponding LCP value is in ascending order and the largest value of LCP is no more than 100, so the capacity of the stack doesn't need more than 100.
