# Supplementary material for: Speeding up tandem mass spectrometry-based database searching by longest common prefix
Source: BMC Bioinformatics. 2010 Nov 25;11:577. doi: 10.1186/1471-2105-11-577 (PMC3000425; doi:10.1186/1471-2105-11-577)
Supplement: Additional file 4 — The proof that ABLCP can obtain all non-redundant semi-specific digestion peptides. [file 1471-2105-11-577-S4.PDF]

The proof that ABLCP can obtain all non-redundant semi-specific digestion peptides.

Proof. We suppose that the cleavage site is only the character 'K', the character 'X' indicates any amino acid character and the character '_' indicates any amino acid character except 'K'. The character before '.' means the peptide's previous character. Then there are three types of peptides for semi-specific digestion:

Type 1: K.XXXK

Type 2: _.XXXK

Type 3: K.XXX_

We prove that ABLCP can obtain all non-redundant semi-specific substrings by the following:

1. All of the substrings can be obtained.

The first type and second type of peptides can be expressed together as X.XXXK. Thus, these types of peptides can be obtained by the algorithm GetAllSubStrings with the LCP not adjusted, but a peptide is retained only when its first right amino acid is a cleavage site.

The third type of peptides can be obtained by generating peptides using the algorithm GetAllSpecificSubStrings with the LCP adjusted, but a peptide is retained only when its first right amino acid is not a cleavage site.

Thus, all the substrings can be obtained.

2. No two obtained substrings are the same.

Peptides obtained by algorithm GetAllSubStrings or GetAllSpecificSubStrings are non-redundant, so within the three types, peptides are non-redundant. Furthermore, a peptide of the first or second type is not the same as the one in the third type, because its first right amino acid is not the same. Thus no two obtained substrings are the same.

As described above, all of the substrings can be obtained and are non-redundant, so it is proven that ABLCP can obtain all non-redundant semi-specific substrings. End of proof.
